# Micro-chimerism: from evolution to revolution

**DOI:** 10.1007/s00281-025-01060-x

**Published:** 2025-08-06

**Authors:** Christopher Urbschat, Petra C. Arck

**Affiliations:** 1 Division of Experimental Feto-Maternal Medicine, Department of Obstetrics and Fetal Medicine, University Medical Center Hamburg-Eppendorf, Hamburg, Germany; 2 Hamburg Center for Translational Immunology, University Medical Center Hamburg-Eppendorf, Hamburg, Germany; 3 German Center for Child and Adolescent Health, Hamburg, Germany

Microchimerism refers to the presence of a small number of foreign, mostly conspecific, cells within an individual. Decades ago, the pioneering researcher in this field faced significant skepticism because the prevailing view was that foreign cells could not survive for long periods inside the host. Today, the existence of microchimerism is widely accepted. Nonetheless, the field still lacks a clear quantitative definition, especially in mammals. There is no universally agreed threshold or precise boundary that qualifies as microchimerism [1]. Additionally, distinguishing microchimerism from other types, such as ‘macrochimerism’ or ‘multichimerism,’ remains difficult. Although its functional role is largely an unresolved mystery, recent studies show that chimerism plays an essential role during immune [2] and neurocognitive development [3]. Here, we compile a comprehensive collection of articles highlighting recent advances across different areas of chimerism research and summarize the known types of chimerism and their modes of acquisition.

The rejection of foreign cells is typically attributed to immune responses against alloantigens on the graft. This aligns with a basic immunological concept of distinguishing between “self” and “non-self” by the host’s immune system [4]. However, in the late 1990s, Polly Matzinger introduced the “danger model,” suggesting that immune reactions can also be triggered by damage or danger signals, which challenges the simple self/non-self classification [5].

Similarly, researchers studying microchimerism describe the notions of “self” and “non-self” as flexible and dependent on context, rather than static responses.

The existence of chimerism and its ability to evade rejection by both innate and adaptive immune responses seems to have developed through evolution. Rinkevich & Goulet emphasize the diverse aspects of chimerism across more than 10 phyla, including protists, plants, vertebrates, and invertebrates [6]. They suggest that chimerism plays a crucial ecological and evolutionary role, influencing the life history traits of metazoans by merging natural transplantation processes with concepts from evolutionary ecology.

Research on bacteria has provided valuable insights into the acquisition of chimerism. In bacteria, chimerism can appear in various forms, involving the transfer of foreign genetic material through different mechanisms: (i) conjugation, which is transfer via a bridge-like connection between cells [7]; (ii) transduction, involving DNA transfer via bacteriophages [8]; and (iii) transfer through shedding bacterial outer membrane vesicles (OMVs) [9]. Studies on corals have expanded this understanding and offered key insights, particularly regarding how chimerism is distributed. In many coral species, chimerism results from tissue fusion between neighboring colonies [10], which can lead to different spatial distributions within the organism. When both individuals’ biological material is equally present in the soma, it is called mixed chimerism. Sectorial chimerism occurs when each individual occupies specific areas within the organism without mixing their genetic material. In entangled chimerism, one individual dominates certain fragments, such as branches [11]. The occurrence and expression of chimerism in marine organisms, including corals, is a fascinating area, as it provides unique benefits like increased competitive abilities, enhanced fitness, and greater ecological invasiveness [6].

Most animals develop from the fusion of a sperm and an egg cell, creating a single cell with combined DNA. As development progresses, all cells except germline cells contain two DNA sets—one from each parent—ensuring uniform genetic information across cells. In ants and some insects like wasps and bees, only females have body cells with two DNA sets. Males, often originating from unfertilized eggs, usually have only one set of chromosomes. Notably, recent research shows that male yellow crazy ants carry two DNA sets: males have R/W genomes, while queens have R/R genomes. Interestingly, R and W cells are unevenly distributed in males; R cells dominate body tissues, and W cells are more common in sperm. If a W sperm fertilizes an egg, the result is a worker; if an R sperm does, a queen forms. This is the first report of reproductive bias through chimerism. The W genome’s chimerism may confer two fitness benefits: preventing syngamy to spread W and the apparently selfish overrepresentation of W in germline cells. Both are likely influenced by opposing selection pressures related to producing queens, workers, and males from a single genome [12]. A similar pattern has been observed in *Pogonomyrmex* harvester ants [13], though it remains unclear whether this reproductive control via chimerism is unique to ants or common in other insects.

In human medicine, organ and tissue transplantation stands out as a major achievement. It clearly causes artificially induced chimerism, since donor and host cells must coexist within the recipient organism.

The success of transplantation relies on preventing transplant rejection. Currently, this is achieved through long-term immunosuppressive drug use, which can lead to side effects such as increased infection risk, cancer, drug toxicity, and cardiovascular and metabolic diseases [14]. Modern immunoengineering strategies aim to circumvent rejection. Ohm and colleagues, as well as Kruchen and colleagues, suggest that microchimerism research has important translational applications in transplantation medicine, including organ [15] and stem cell transplants [16]. It is already understood that a mismatch in non-inherited maternal antigens (NIMA) can positively influence transplantation outcomes. Blood and umbilical cord blood banks now incorporate NIMA-mismatch typing. Recent theories propose the existence of a chimeric stem cell niche dominated by a single cell population, implying that birth order affects mother-child transplants, with sons likely being the optimal donor for their mothers.

Plant transplantation is much less understood than medical transplantation, yet it holds significant importance. In 1674, Pietro Nati, a gardener from Florence, was the first to report a chimeric plant after noticing an adventitious scion growing from the grafting site between a bitter orange and citron [17]. This chimera displays features reminiscent of both citrus ancestors and has sparked considerable interest and discussion among scientists. Today, grafting—also known as inducing chimerism in plants—remains a vital technique for artificial vegetative propagation. It is used for species preservation and to make plants accessible in various habitats, especially where soil conditions are unsuitable for certain root systems [18].

During pregnancy, there is a bidirectional transfer of cells between mother and fetus. Some maternal cells remain in the offspring into adulthood, known as maternal microchimeric cells, while remnants of previous pregnancies persist in the mother for life, termed fetal microchimerism. Pregnancy is unique because it is the only natural occurrence of chimerism in humans and other mammals. As a result, research into pregnancy-induced chimerism is increasingly important across various fields such as transplantation medicine, autoimmunity, cancer, and inflammation resolution [19]. In women with multiple pregnancies, fetal microchimeric cells from each pregnancy are retained, along with maternal cells acquired during the period of fetal development of the pregnant women themselves—this phenomenon is called transgenerational chimerism [20]. Pregnancy-acquired chimerism has significant implications for understanding immune tolerance and autoimmunity, especially related to organ transplants and autoimmune diseases, which more commonly affect women. Lee and Lambert discuss how pregnancy-related chimerism may influence autoimmunity, suggesting that the accumulation of chimeric cells and their mismatch in HLA molecules might contribute to autoimmune diseases through self-peptide presentation [21]. Interestingly, even men and women who have never been pregnant may carry chimeric cells inherited from their mother or older siblings. Therefore, the relationship of HLA molecules within families warrants further study to understand chimerism’s role in autoimmunity and healthy alloimmunity.

Kroneis and colleagues suggest several possible mechanisms for transferring chimeric cells at the feto-maternal interface. These include HLA-compatibility and inflammatory processes such as placental inflammation, chronic chorioamnionitis, and labor-induced inflammation [22]. These mechanisms can be influenced by various inflammatory signals, which are also associated with other pregnancy complications [23].

Further, Parmeggiani and colleagues discuss the impact of pregnancy-acquired microchimerism on cancer progression in parturient females and whether it is protective or promotive [24].

New methodological approaches are now accessible or close to being available, such as digital droplet PCR, where primers are continually refined and the scope of detectable targets expands. Additionally, sequencing methods not only enable detection but also provide insights into cell phenotypes and functions [25, 26]. Consequently, applying research findings on microchimerism in translational settings will allow for quicker and more sensitive detection of these cells, ideally through blood samples [27].

**Fig. 1:**
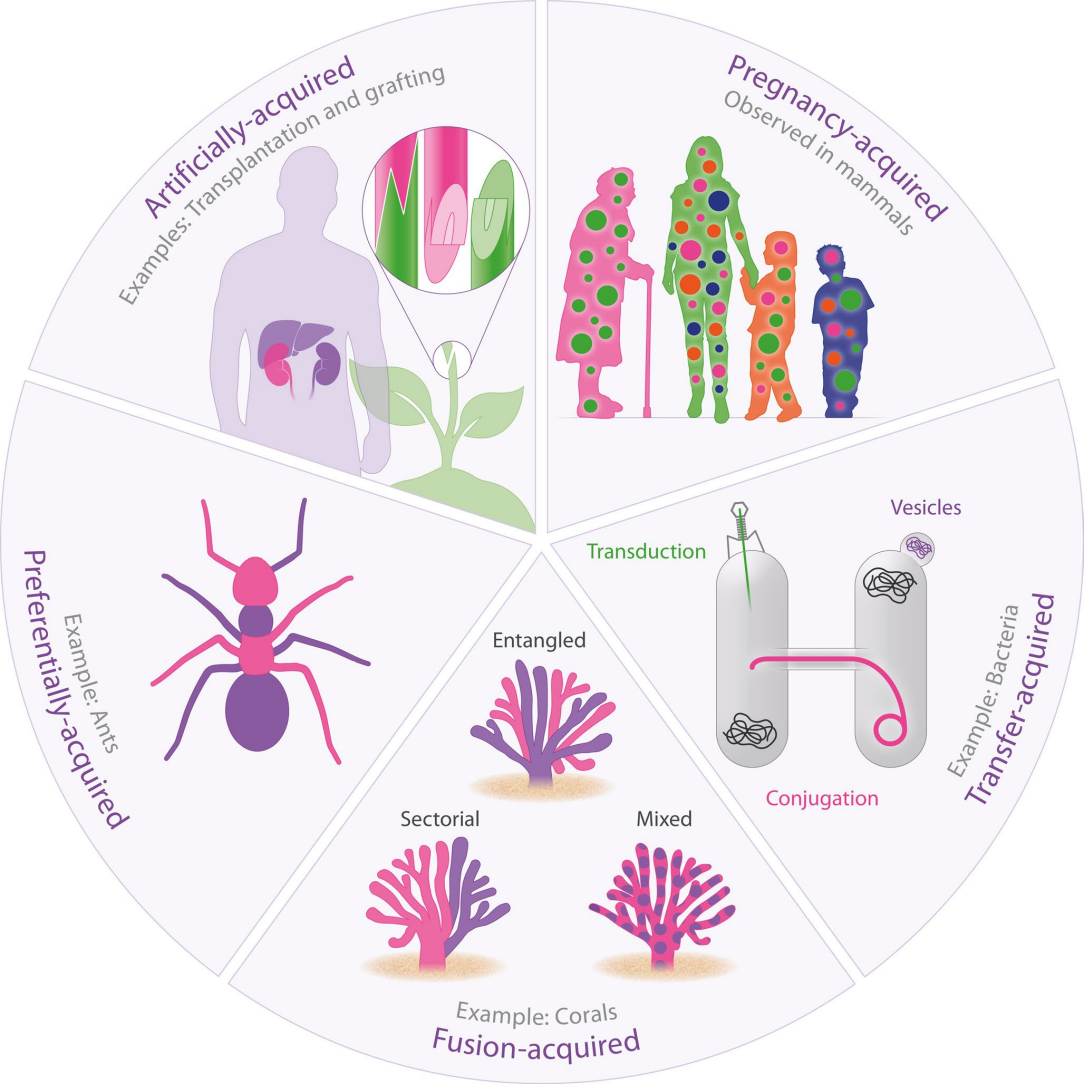
Types of chimerism and their modes of acquisition

This collection of articles highlights the diversity, acquisition and biological roles of chimerism (Fig. 1). It offers a comprehensive review of how chimerism functions across different species, synthesizing various perspectives to explore the complex interactions between chimeric cells and the host organism, along with their effects on health and disease. Addressing this highly conserved and intricate biological phenomenon, these articles are likely to inspire further research, encourage collaboration, and potentially revolutionize the development of new diagnostic tools and targeted therapies, providing insights into the future of gene editing.
